# Simpson’s Paradox in Clinical Research: A Cautionary Tale

**DOI:** 10.3390/jcm12041633

**Published:** 2023-02-18

**Authors:** Stefanos Bonovas, Daniele Piovani

**Affiliations:** 1 Department of Biomedical Sciences, Humanitas University, Pieve Emanuele, 20072 Milan, Italy; 2 IRCCS Humanitas Research Hospital, Rozzano, 20089 Milan, Italy

The word paradox comes from the Greek *paradoxon*, meaning something that was contrary to, or contradicted, common sense. Paradoxes are marvels of the human mind, typically formulated at the intersection of logic and philosophy. Nowadays, one of the most fascinating and intriguing paradoxes in statistical science is Simpson’s paradox, which carries significant implications for medical research and practice [1].

Simpson’s paradox is a statistical phenomenon in which an observed association between two variables at the population level (e.g., positive, negative, or independent) can surprisingly change, disappear, or reverse when one examines the data further at the level of subpopulations. It was first pointed out by Pearson (1899) [2] and Yule (1903) [3], but it was Simpson’s paper (1951) that demonstrated how combining contingency tables can lead to paradoxical conclusions [4]. Simpson’s paradox arises from the combination of an overlooked confounding variable and a disproportionate allocation of that variable. There are several exciting examples in the fields of epidemiology and clinical research, where understanding the paradox is essential for drawing proper conclusions regarding the effectiveness of treatments, the effect of exposure to risk factors on medical hazards, and health policy decision-making.

A well-known demonstration of Simpson’s paradox comes from a study comparing open surgery vs. percutaneous nephrolithotomy to treat kidney stones [5,6]. Table 1 summarizes the success rates of these two approaches, also stratified by stone size. The paradox is that open surgery is associated with higher success rates for small stones (93.1% vs. 86.7%; Relative Risk (RR) = 1.07) and large stones (73.0% vs. 68.8%; RR = 1.06), while percutaneous nephrolithotomy appears to be more effective than open surgery when the stone diameter is not taken into account (i.e., aggregate analysis: 78.0% vs. 82.6%; RR = 0.94; Table 1).

The reason behind this surprising reverse of the direction of the association is that the probability of having one treatment or the other depended on the size of the stones (confounding variable). Most patients with kidney stones of a diameter smaller than 2 cm (i.e., 270/357 or 75.6%) had percutaneous nephrolithotomy, while the majority of patients with stones of diameter larger than 2 cm or with multiple stones (i.e., 263/343 or 76.7%) had open surgery (i.e., disproportionate allocation of the confounding variable).

Another example of the paradox comes from the hospital epidemiology field [7,8]. Table 2 presents surveillance data from eight Dutch hospitals regarding urinary tract infections (UTI) in patients receiving and patients not receiving antibiotic prophylaxis. The paradox here is that antibiotic prophylaxis is associated with a lower rate of UTI in the aggregate analysis of all the hospitals (UTI: 3.3% vs. 4.6%; RR = 0.71); however, when one stratifies the hospitals into two groups depending on whether the rate of UTI is lower or higher than 2.5%, the association previously seen now reverses both in the hospitals of low-incidence (UTI ≤ 2.5%: 1.8% vs. 0.7%; RR = 2.59) and in the hospitals of high-incidence (UTI > 2.5%: 13.3% vs. 6.5%; RR = 2.03; Table 2).

The stratum-specific data reveal the opposite effect of what is seen in the complete, unstratified set of data. The reason behind this paradoxical reverse of the direction of the association was the fact that the percentage of patients receiving antibiotic prophylaxis varied significantly between the low-incidence hospitals (i.e., 1113/1833 or 60.7%) and the high-incidence hospitals (i.e., 166/1686 or 9.8%). In other words, the variable distinguishing the strata in Table 2 (being a patient in a certain hospital) acts as a confounder because it is associated both with antibiotic prophylaxis (exposure variable) and with UTI (outcome variable).

We can also use a more recent example of Simpson’s paradox, from the COVID-19 era, to illustrate its implications in health policy decisions. In 2020, early epidemiologic data showed that the case fatality rate for COVID-19 was higher in Italy than in China overall. However, this crude analysis proved to be confounded by age (because the distribution of COVID-19 cases across age groups differed significantly between the two countries). Analysis of the data by age strata revealed that within every age group, the case fatality rate was actually higher in China than in Italy [9].

Simpson’s paradox is a compelling demonstration of why rigorous and thoughtful statistical analyses are needed in clinical research, and how easy it is to draw the wrong conclusions when relying solely on intuition. It reminds us to think critically about data, especially data from non-randomized research; interpret with caution every association achieving statistical significance [10], with double caution if the finding was unexpected; and carefully examine for confounding factors, because overlooking such factors can lead to erroneous conclusions and harmful consequences for medical research and practice. Clinical investigators are strongly encouraged to obtain consultation and collaboration from biostatisticians and research methodologists early in the development and conduct of their studies because as Sir Ronald Fisher, the founder of modern statistics, remarked: *“To call in the statistician after the experiment is done may be no more than asking him to perform a postmortem examination: he may be able to say what the experiment died of“* [11].

## Figures and Tables

**Table 1:** Success rate in removing kidney stones by treatment method * (data from Charig [5]).

	Treatment of Kidney Stones		
	*Open Surgery*	*Percutaneous Nephrolithotomy*	
Stone diameter < 2 cm	81/87 (93.1%)	234/270 (86.7%)	RR = 1.07
Stone diameter ≥ 2 cm	192/263 (73.0%)	55/80 (68.8%)	RR = 1.06
All stones (aggregate)	273/350 (78.0%)	289/350 (82.6%)	RR = 0.94

* Figures are numbers (%) of patients.

**Table 2:** Rate of urinary tract infections by antibiotic prophylaxis * (data from Reintjes [7]).

	Antibiotic Prophylaxis		
	*Yes*	*No*	
Low-incidence hospitals	20/1113 (1.8%)	5/720 (0.7%)	RR = 2.59
High-incidence hospitals	22/166 (13.3%)	99/1520 (6.5%)	RR = 2.03
All hospitals (aggregate)	42/1279 (3.3%)	104/2240 (4.6%)	RR = 0.71

* Figures are numbers (%) of patients.
